# Organizing pneumonia after stereotactic ablative radiotherapy of the lung

**DOI:** 10.1186/1748-717X-7-123

**Published:** 2012-08-01

**Authors:** Taro Murai, Yuta Shibamoto, Takeshi Nishiyama, Fumiya Baba, Akifumi Miyakawa, Shiho Ayakawa, Hiroyuki Ogino, Shinya Otsuka, Hiromitsu Iwata

**Affiliations:** 1 Department of Radiology, Nagoya City University Graduate School of Medical Sciences, Nagoya, Japan; 2 Department of Public Health, Nagoya City University Graduate School of Medical Sciences, Nagoya, Japan; 3 Department of Radiology, Social Insurance Chukyo Hospital, Nagoya, Japan; 4 Department of Radiology, Nagoya Daini Red Cross Hospital, Nagoya, Japan

**Keywords:** Stereotactic ablative radiotherapy (SABR), Organizing pneumonia, Lung cancer, Radiation pneumonitis

## Abstract

**Background:**

Organizing pneumonia (OP), so called bronchiolitis obliterans organizing pneumonia after postoperative irradiation for breast cancer has been often reported. There is little information about OP after other radiation modalities. This cohort study investigated the clinical features and risk factors of OP after stereotactic ablative radiotherapy of the lung (SABR).

**Methods:**

Patients undergoing SABR between 2004 and 2010 in two institutions were investigated. Blood test and chest computed tomography were performed at intervals of 1 to 3 months after SABR. The criteria for diagnosing OP were: 1) mixture of patchy and ground-glass opacity, 2) general and/or respiratory symptoms lasting for at least 2 weeks, 3) radiographic lesion in the lung volume receiving < 0.5 Gy, and 4) no evidence of a specific cause.

**Results:**

Among 189 patients (164 with stage I lung cancer and 25 with single lung metastasis) analyzed, nine developed OP. The incidence at 2 years was 5.2% (95% confidence interval; 2.6-9.3%). Dyspnea were observed in all patients. Four had fever. These symptoms and pulmonary infiltration rapidly improved after corticosteroid therapy. Eight patients had presented with symptomatic radiation pneumonitis (RP) around the tumor 2 to 7 months before OP. The prior RP history was strongly associated with OP (hazard ratio 61.7; *p* = 0.0028) in multivariate analysis.

**Conclusions:**

This is the first report on OP after SABR. The incidence appeared to be relatively high. The symptoms were sometimes severe, but corticosteroid therapy was effective. When patients after SABR present with unusual pneumonia, OP should be considered as a differential diagnosis, especially in patients with prior symptomatic RP.

## Introduction

Organizing pneumonia (OP), so called bronchiolitis obliterans organizing pneumonia, is a rare interstitial lung disease having unique clinical, radiological and pathological characteristics [1-3]. Typical OP patients present with dyspnea, cough, and fever that have been developing over a few weeks. Dramatic improvement is achieved by corticosteroids, but relapses occur frequently after corticosteroid therapy is tapered or stopped. Since Crestani et al. [4]. reported OP after postoperative irradiation for breast cancer in 1998, several reports have described the features, which are different from those of radiation pneumonitis (RP) [5-10]. The infiltrates initially appear in the irradiated side of the lung and migrate outside the radiation field. These features lead to the notion that radiation injury may prime the development of OP. However, no reports have demonstrated the relationship between radiation injury and OP, and there is little information about OP after other radiation modalities.

Stereotactic ablative radiotherapy of the lung (SABR), previously called stereotactic body radiotherapy of the lung is a technique to precisely deliver radiation to a targeted tumor. Although surgical resection has been regarded as standard therapy for early stage non-small cell lung cancer (NSCLC) and solitary lung metastases, recent reports on SABR for inoperable patients show minimal morbidity and high local control rates [11-15]. Accordingly, the numbers of patients with lung cancer or solitary lung metastasis treated with SABR have recently increased for the reasons they are medically inoperable or refuse surgery [16].

To the best of our knowledge, OP after SABR has not been reported yet. We herein describe the clinical features of OP after SABR performed at two institutions. The objective of this study was 1) to describe the incidence and characteristics of OP after SABR, and 2) to investigate risk factors.

## Method

### Patients

Nearly all data used in this study were obtained from patients enrolled in multi-institutional protocol-based SABR studies [13,14,17-19]. Between April 2004 and May 2010, 210 patients entered the SABR studies at two institutions (Nagoya City University Hospital and Nagoya Daini Red Cross Hospital). All these patients had T1N0M0 or T2aN0M0 stage NSCLC according to the 7^th^ edition of TNM staging at diagnosis, or single lung metastasis. Local recurrences after surgery less than 5 cm in diameter were included. The patients’ medical records, radiotherapy documents and images were reviewed. Of the 210 patients, seven had undergone prior chest irradiation and 14 had interstitial lung disease diagnosed based on clinical symptoms, Kerbs von Lungren (KL)-6 elevation, computed tomography (CT) findings and/or biopsy [18]. These patients were excluded from analysis because it is hard to distinguish RP after prior radiotherapy or exacerbation of interstitial pneumonia from OP after SABR. Thus, 189 patients were analyzed. The characteristics of these patients are shown in Table 1. Four patients had a previous diagnosis of rheumatoid arthritis. All patients provided written informed consent. This study was approved by the institutional research and ethics committees (Nagoya City University Hospital, No. 532 and Nagoya Daini Red Cross Hospital, IRB20110125-1). 

**Table 1:** Patient characteristics and SABR parameters

		***Patient Number***	***(%)***
***Male/Female***		120/69	(63.5/26.5)
***Institution***			
	*Nagoya City University Hospital*	175	(92.6)
	*Nagoya Daini Red Cross Hospital*	14	(7.4)
***Disease stage***			
	*T1N0M0*	110	(58.2)
	*T2aM0N0*	47	(24.9)
	*Metastasis*	25	(13.2)
	*Local recurrence*	7	(3.7)
***Prescribed dose (Gy/fractions)***			
	*52/4*	50	(26.5)
	*50/4*	26	(13.8)
	*48/4*	97	(51.3)
	*44/4*	3	(1.6)
	*36/2*	4	(2.1)
	*34/2*	6	(3.2)
	*24/2*	1^*^	(0.5)
	*54/6*	2	(1.1)
***Age (years)***		Median 76 (Range 16 - 89)	
***Planning target volume (cm***^***3***^***)***		52.7 ± 29.1^**^	
***Mean lung dose (Gy)***		4.5 ± 1.8^*^	
***V20 Gy***^†^***(%)***		6.7 ± 3.1^*^	

^*^This patient could not complete the planned protocol of 48 Gy in 4 fractions.

^**^ Mean ± standard deviation.

^†^ V20 Gy, lung volume covered with 20 Gy or more.

### Treatment methods

Our methods for treatment planning were described in detail previously [13,14]. The clinical target volume (CTV) was defined as the visible gross tumor volume. The CTV was expanded to the internal target volume (ITV), considering respiratory motion of the CTV. The ITV was extended by 5 to 10 mm to represent the planning target volume (PTV). Irradiation to the PTV was delivered using three coplanar and four noncoplanar static ports. SABR was delivered by a linear accelerator with 6-megavolt photons.

### Prescription dose

The prescribed dose was delivered to the isocenter depending on the maximum diameter. On the protocols, the dose was 48 or 50 Gy in 4 fractions for both NSCLC and metastasis with a longest diameter of 1.5 to 3 cm, and 52 Gy in 4 fractions for tumors larger than 3 cm. Smaller NSCLC (< 1.5 cm) were treated with 44 or 48 Gy in 4 fractions and smaller metastases (< 1.5 cm) were irradiated at 34 or 36 Gy in 2 fractions. Beginning in January 2009, concurrent chemotherapy with TS-1 (80 mg/m [2] per day) was administered to 4 patients with T2a NSCLC over 28 days. Chemotherapy for other cancers such as colorectal cancer and small cell lung cancer was carried out in 3 patients during SABR.

All patients, except for two initial cases treated with 54 Gy in 6 fractions, were treated on this protocol (Table 1). Most patients (175/189) received 48 to 52 Gy in 4 fractions. The Eclipse AAA system or Pinnacle (3) collapsed cone convolution were used as the dose calculation algorithm. Lung volume covered with 20 Gy or more (V20 Gy), mean lung dose and PTV (cm [3]) were evaluated on the treatment planning workstation.

### Diagnosis of OP after SABR

The criteria for the diagnosis of OP after SABR were defined by reference to previous criteria in breast cancer studies: [4,6-10] 1) mixture of patchy and ground-glass opacity, 2) general and/or respiratory symptoms lasting for at least 2 weeks, 3) radiographic lesion in the lung volume receiving less than 0.5 Gy, and 4) no evidence of a specific cause. Most patients in this study were elderly or had severe comorbid disease. Thus, biopsy could not be included in these criteria in accordance with the previous breast cancer studies. The diagnosis was determined by two physicians (T. M. and A. M.) using the 2-item questionnaire.

### Follow-up studies

Patients were followed at 1- to 2-month intervals after SABR. Physical examinations, chest radiographs or CT, and laboratory tests were routinely performed at each visit. Toxicity was evaluated using Common Terminology Criteria for Adverse Events Version 4. The follow-up period after SABR ranged from 2 to 85 months (median, 26 months).

### Statistical analyses

The primary endpoint of the study was the incidence of OP. The time was measured from the date of starting SABR. First, the cumulative incidence of OP was calculated by accounting for death or other thoracic irradiation as competing risks [20,21]. To select variables used in the multivariate analysis, the OP incidence was compared with the Gray test for equality among levels of covariates: age, sex, site, stage, dose, chemo, rheumatoid arthritis, PTV (cm [3]), V20 Gy and mean lung dose [22]. Variables for which *p*-value was < 0.20 in the Gray test were included in the multivariate regression analysis, using the semiparametric proportional hazards model of Fine and Gray [20,23]. The subdistributional hazard ratio (HR) for a categorical covariate was the ratio to subdistribution hazards for the actual group with respect to the baseline, with all other covariates being equal [20,23].

**Figure 1:**
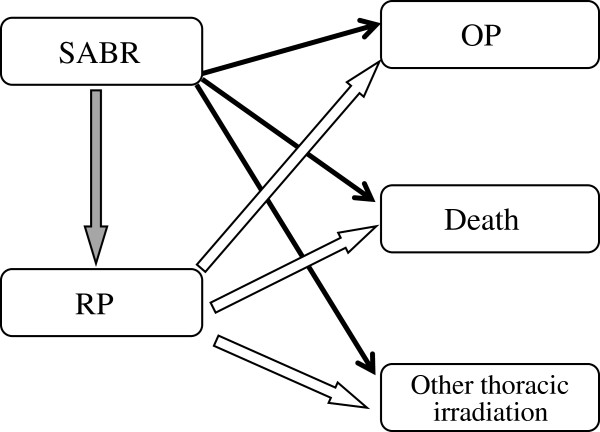
**Five clinical states and transitions in the multi-state model.** The multi-state model was developed with five clinical states: the index SABR, RP, OP, death and other thoracic irradiation. Each arrow denotes the transition between two clinical states. A transition to RP (gray arrow) was considered as an intermediate event.

Second, to investigate the impact of the RP incidence as a time-dependent covariate, the multi-state model was developed by five clinical states (Figure 1) [24]. The transitions from SABR to either OP incidence, death, or other thoracic irradiation are modeled by a competing risk model. Thus, this multi-state model generalizes the competing risk model by including an intermediate event of RP. We assumed that two transitions (Figure 1, black arrows and white arrows) had common baseline hazards, respectively. We introduced a time-dependent covariate that indicated whether or not RP has already occurred. For a transition from SABR to OP, the value of this covariate equals zero, while for a transition from RP to OP, the value equals one. In this analysis, we also included variables used in the Fine and Gray analysis. A Kappa agreement score was calculated to evaluate agreement of two physicians. 

Proportional hazard assumptions for all variables included in the univariate and multivariate models were checked by inspection of the Schoenfeld’s residuals. All statistical tests were two-sided. These analyses were implemented in the R package vdc, cmprsk [20,21]or package mstate [25]. All analyses were performed in R version 2.13.0 for Windows [26].

## Results

### Clinical courses of patients with OP after SABR

**Figure 2:**
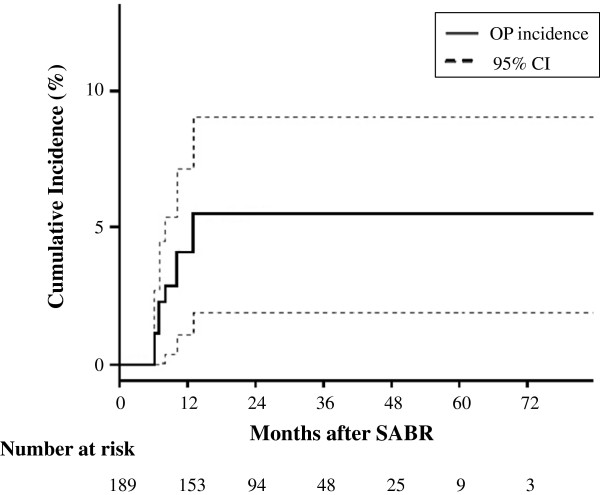
**The incidence of OP after SABR.** CI denotes confidence interval.

Nine patients developed OP at 6–16 months after SABR. The incidences of OP were 4.0% (95% confidence interval: 1.7-7.6%) and 5.2% (2.6-9.3%) at 1 and 2 years, respectively (Figure 2). A Kappa agreement score was 1.0. Opacity outside radiation ports was observed in 2 patients. However, they were asymptomatic, so we did not regard them as OP patients.

**Figure 3:**
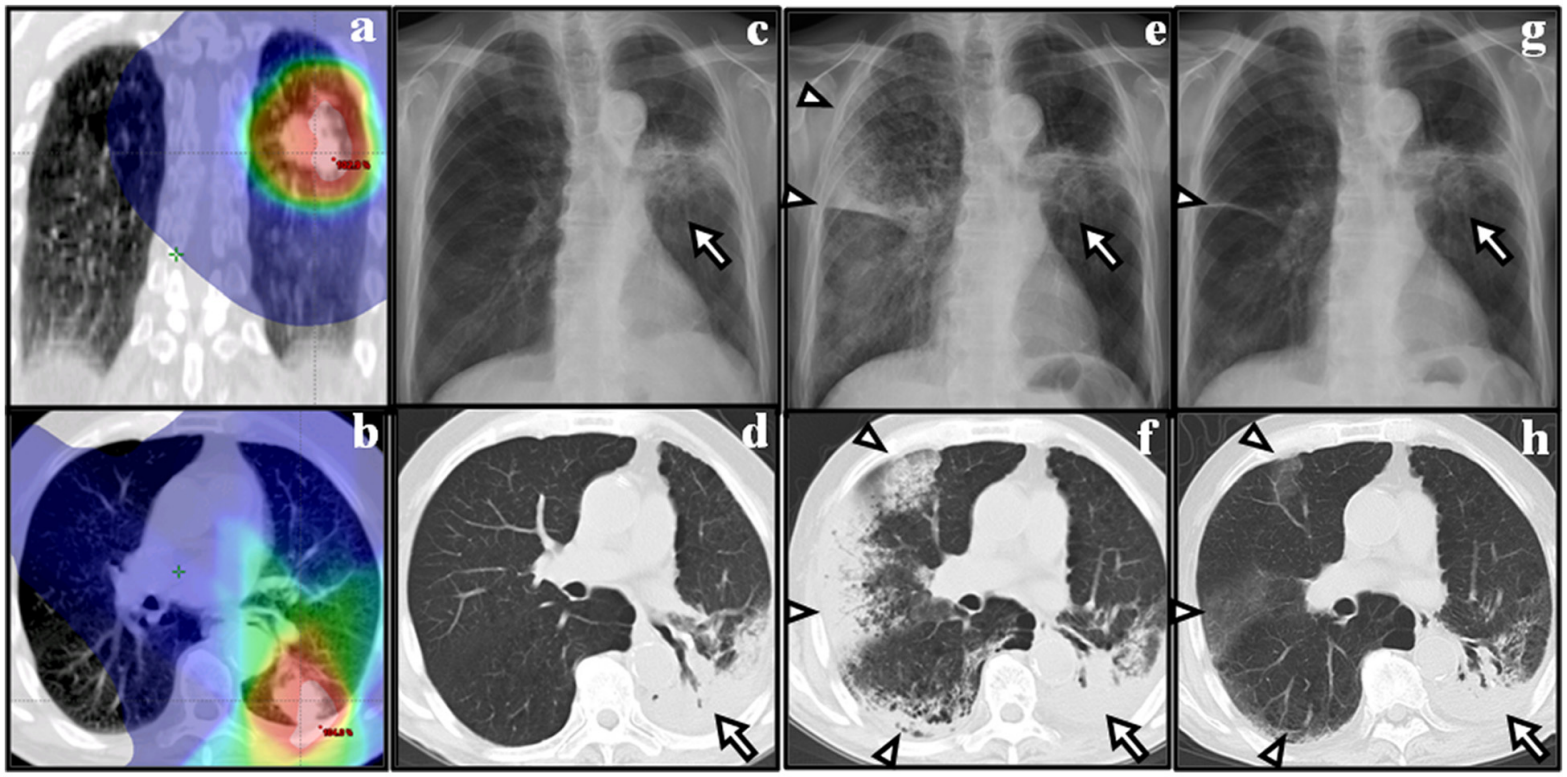
**Chest CT, radiograph and dose distribution of SABR of an 82-year-old patient.** Blue represents areas receiving more than 0.5 Gy (Figure 3**a**, **b**). The patient presented with RP around the tumor 8 months after SABR (Figure 3**c, d** arrow). OP occurred 3 months after RP resolution (Figure 3**e**, **f** arrow head). Opacity around the tumor did not increase significantly (Figure 3**e**, **f**, arrow). After administration of 5 mg prednisone, symptoms and radiological findings improved (Figure 3**g**, **h**).

The clinical features of OP patients are shown in Table 2. An example of OP is shown in Figure 3. Two of the nine were nonsmokers. Three had emphysema. No patient had a history of allergy-related symptoms or rheumatoid arthritis. All these patients had NSCLC (T1N0M0, 4 patients; T2aN0M0, 5 patients) and were treated with SABR alone. The median PTV (cm [3]), V20Gy and mean lung dose were 74.3 cm [3] (range: 42.6-102.1), 7.5% (3.6-9.7%) and 5.5 Gy (3.6-10.1 Gy), respectively, in these patients. Eight of the 9 patients had experienced symptomatic RP around the tumor within 3 to 9 months after SABR. OP in the opposite lung and outside ports appeared 2 to 7 months after the RP. Opacity around the tumor did not increase in any patient. Screening examinations for infectious diseases were negative. All OP patients presented with dyspnea (Grade 2 in 4 patients and Grade 3 in 5). Of the 9 patients, 5 had Grade 3 general fatigue, 5 had fever (≥ 37.5 degrees Celsius) and/or persistent cough, 3 had sputum and 1 had chest pain. C-reactive protein (CRP) elevation (≥ 5.0 IU/ml) was observed in 6 patients. KL-6 elevation (≥ 500 U/ml) was observed in 4 of the 9 patients. Elevation of white blood cell count (> 10,000/μl) was observed in only 2 patients. Prednisone (0.5-1 mg/kg) was administered to 5 of the 9 patients and their symptoms and pulmonary infiltrates were improved within 1 to 4 weeks. Prednisone treatment was tapered over 4 to 8 weeks. Antibiotics were administered to 2 patients without steroids and 2 patients did not receive any treatment at all. In these cases, the chest radiograph findings and symptoms improved gradually within 4 to 16 weeks. 

**Table 2:** Clinical characteristics of OP patients

***No.***	***Age***	***Sex***	Dose (Gy/4 fr^*^)	***Toxicity grade (CTCAE ver4)***			***CRP (IU/ml)***	***KL-6 (U/ml)***^**†**^	***Treatment after first OP***
				***Dyspnea***	***Fever***	***Fatigue***			
**1**	62	M	52	2		2	5.7	813	
**2**	80	M	48	2		3	0.9	456	
**3**	79	M	48	3	1	2	22.7	558	PSL^**^ + Abx^††^
**4**	71	F	52	2	1	2	4.0	299	Abx
**5**	76	M	52	2		2	1.1	414	PSL
**6**	82	M	52	3	2	3	21.0	653	PSL + Abx
**7**	83	M	52	3	2	3	10.6	231	Abx
**8**	79	M	50	3	2	3	5.8	531	PSL + Abx
**9**	83	F	50	3		3	5.9	314	PSL + Abx

^*^fr = fractions. ^**^PSL = Prednisone. ^††^Abx = antibiotics.

^†^ Normal range for KL-6 is ≤ 500 U/ml in the institutions.

CRP and KL-6 data represent the maximum level during the initial OP course. Relapse of OP occurred in patients No. 6-9.

Relapse of OP occurred in 4 patients (Table 2, patient number 6–9). They received treatment similar to that used for first OP. One patient needed intubation for Grade IV dyspnea. One and two episodes of relapse occurred in 2 patients each. One patient suffered first relapse during prednisone tapering. Increasing the prednisone dose was effective in these patients. At latest follow-up, none of the 4 patients had clinical symptoms, although 3 of them were still receiving prednisone at a dose of 5 mg, and chest roentgenograms revealed small residual opacities and linear densities in all patients.

### Multivariate analyses of factors associated with OP after SABR

**Figure 4:**
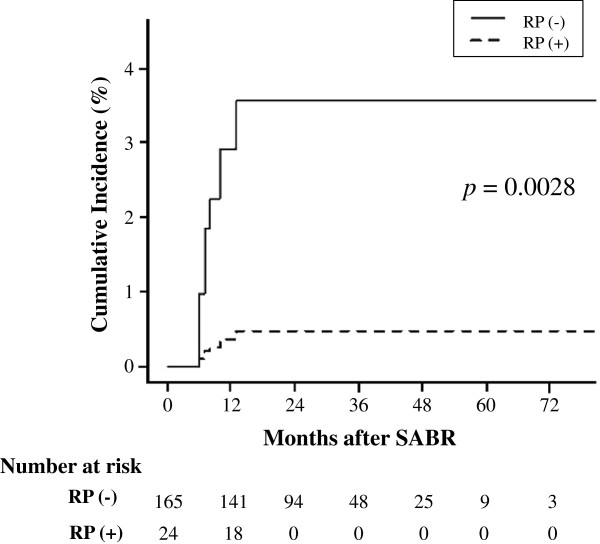
**Incidence of OP after SABR in patients with (+) and without (−) prior symptomatic RP in multi-state model.** CI denotes confidence interval.

In the Gray test, risk factors with a *p*-value < 0.20 were PTV (cm [3]) (*p* = 0.039) and dose (*p* = 0.19). In the Fine and Gray analysis, no factor showed statistical significance (Table 3). We then investigated the impact of the RP incidence using a multi-state model (Figure 1). In this analysis, we also included variables used in the Fine and Gray analysis. This analysis showed that only prior symptomatic RP was significantly associated with OP after SABR (HR: 61.7: 95% confidence interval: 4.1-928.1, *p* = 0.0028) (Figure 4). 

**Table 3:** Fine and Gray regression analysis

	***Patient number***		***HR***	***(95% CI)***	***p-value***
	***Total***	***(with OP)***			
***Dose (Gy/fractions)***					*0.4*
* 48/4*	97	(2)	1		
* 50/4*	26	(2)	3.21	(0.47 -21.86)	
* 52/4 and other*	66	(5)	2.56	(0.57 -11.53)	
***PTV (cm***^***3***^***)***					*0.4*
* 4.5 -44.2*	93	(1)	1		
* 44.2 -71.6*	48	(3)	5.98	(0.65 -54.7)	
* 71.6 -192*	48	(5)	7.46	(0.84 -66.3)	

## Discussion

We have described the clinical features of OP after SABR. They were quite similar to those of OP after postoperative irradiation for breast cancer and clearly differed from those of RP [4,6-10,27,28]. To the best of our knowledge, this report represents the first cohort study on OP after SABR. This study suggests some clinical implications for SABR. First, OP after SABR occurred in 9 out of 189 (4.8%) in the present study, while previous reports described OP incidences after postoperative irradiation for breast cancer as 1.9% to 2.5%. Our incidence of OP after SABR appears to be relatively high, and this may be related to the relatively large volume of the lung irradiated at SABR as compared with postoperative irradiation for breast cancer. The symptoms were sometimes severe, compared to those of patients developing OP after postoperative irradiation for breast cancer [8]. Secondly, most OP occurred a few months after RP in this study. A very strong association between prior RP history and OP was also shown statistically. All OP developed within 16 months after SABR. Thus, when a patient after SABR presents with unusual pneumonia, OP should be considered in the differential diagnosis. Patients after SABR should be carefully followed up for at least 2 years, especially when RP is symptomatic. Finally, the clinical features of OP after radiation could differ from those of RP. Patchy consolidation and ground-glass opacity are frequently observed in both of these diseases. [6-10] In addition, bilateral RP outside the radiation port is occasionally observed. However, RP in the contra-lateral lung is less marked than that around the tumor [6-10]. In OP patients, opacity appeared dominantly outside radiation ports a few months after the RP improvement as was the case in our study. At the same time, worsening of RP around the tumor was not observed. Future studies on SABR would prove OP after SABR as a complication separate from RP.

The mechanism of OP after radiation has not been understood well. This study offers some clues and insights. Most OP patients had a history of prior RP around the tumor in this study. This result would indicate that radiation injury plays an important role in the development of OP after SABR. Previous studies reported that radiation can directly induce Fas expression in cells and activate inflammatory cells expressing Fas-ligand (macrophages and T cells) [29-31]. Martin et al. [32]. reported that unilateral thoracic irradiation induced elevation of lymphocytes in bilateral lungs. In OP model mice, neonatal thymectomy inhibited the development of OP [33]. Mice with blockage of the Fas/Fas-ligand apoptotic pathway could not develop OP [34]. Thus, OP development may require the T-cell and Fas/Fas-ligand pathways, which can be activated by radiation in OP after SABR.

Interestingly, OP after SABR occurred outside the lung volume receiving less than 0.5 Gy. Although a radiation dose less than 0.5 Gy is generally too low to induce cell death directly, it is sufficient to induce a bystander effect [35,36]. The bystander effect is defined as a non-targeted phenomenon induced by low dose radiation, and includes mutations, genetic instability, formation of micronuclei and apoptosis [35]. The mechanisms involve inflammatory cells and biochemical and molecular signals, for example, tumor necrosis factor-alpha, nitric oxide and superoxide. Tumor necrosis factor-alpha enhances Fas mediated apoptosis in lung epithelial cells [37]. In this study, no significant relationships between OP and radiation intensity, such as prescribed doses were observed. This result is consistent with previous reports on bystander effects; in most studies, the magnitude had no simple relationship with dose [35,38]. Saturation of responses may occur due to a limit of how much signal can be produced by the irradiated cells. Therefore, if an interaction between the bystander effect and activated T-cells and Fas/Fas-ligands by direct radiation plays an important role in OP after SABR, a dose response model may not apply to OP after radiation.

This study had some limitations. First, the definition of OP in this study was determined by the clinical course and radiological findings with reference to previous criteria in breast cancer studies. Neither biopsy nor bronchoalveolar lavage could be performed in this study, because the OP patients were too frail or too old to undergo these invasive examinations, or refused them. Generally, characteristics of OP on CT images are well correlated with histological characteristics [1,3,39]. OP is characterized by mixture of patchy and ground-glass opacity in subpleural or peribronchial areas on CT. These findings correspond to the histological findings of mild inflammation and polypoid plugs of loose organizing connective tissue with or without endobronchiolar intraluminal polyps. The architecture of the lung is preserved. Although most of the differential diagnoses like other malignancies and pulmonary infectious diseases could be excluded in the appropriate clinical context, it is possible that the true incidence of OP was overestimated [3,39]. Secondly, we could not completely exclude other causes, especially lung cancer. Malignancy sometimes induces OP [3,40]. Thus, we started a multi-cohort study to evaluate OP incidences between a surgery group and an SABR group.

In conclusion, this report is the first cohort study to describe the incidence and characteristics of OP after SABR. Prior symptomatic RP was strongly associated with OP. The symptoms were sometimes severe and corticosteroids were effective. Patients after SABR should be carefully followed, especially when RP is symptomatic. When patients after SABR present with unusual pneumonia, this disease should be considered in the differential diagnosis.

## Abbreviations

OP: Organizing pneumonia; RP: Radiation pneumonitis; SABR: Stereotactic ablative radiotherapy of the lung; NSCLC: Non-small cell lung cancer; CTV: Clinical target volume; ITV: Internal target volume; PTV: Planning target volume; V20 Gy: Lung volume covered with 20 Gy or more; CRP: C-reactive protein; KL-6: Kerbs von Lungren-6; CT: Computed tomography; HR: Hazard ratio.

## Competing interests

The authors declare that they have no competing interests.

## Authors’ contributions

TM: contributed to designing the search strategy, data abstraction, data analysis, data interpretation, drafting of the manuscript, revising the article critically for important intellectual content, and approving the final version of this manuscript. YS: contributed to study designing the search strategy, data analysis, revising the article critically for important intellectual content, and approving the final version of this manuscript. TN: contributed to study designing the search strategy, data analysis, revising the article critically for important intellectual content, and approving the final version of this manuscript. FB: contributed to study data analysis, revising the article critically for important intellectual content, and approving the final version of this manuscript. AM: contributed to study data analysis, revising the article critically for important intellectual content, and approving the final version of this manuscript. SA: contributed to study data abstraction, revising the article critically for importantintellectual content, and approving the final version of this manuscript. HO: contributed to study data abstraction, revising the article critically for important intellectual content, and approving the final version of this manuscript. SO: contributed to study data abstraction, revising the article critically for important intellectual content, and approving the final version of this manuscript. HI: contributed to study data abstraction, revising the article critically for important intellectual content, and approving the final version of this manuscript.

### Other contributions

The authors wish to thank Drs. Rumi Murata, Takeshi Yanagi, Aiko Nagai, Naoki Hayashi, Shinya Takemoto and Katsura Kosaki and Mrs Hiroshi Fukuma and Kazuhiro Komai for their valuable help in this research.Presented at the 52nd ASTRO annual meeting, 31^st^ October-4^th^ November, 2010, San Diego, California, USA.This work was supported in part by Grants-in-Aids for Scientific Research from the Japanese Ministry of Education, Culture, Sports, Science and Technology.
